# Load Deflection Characteristics of Orthodontic Gummetal^®^ Wires in Comparison with Nickel–Titanium Wires: An In Vitro Study

**DOI:** 10.3390/ma17020533

**Published:** 2024-01-22

**Authors:** Hisham Sabbagh, Mila Janjic Rankovic, Daniel Martin, Matthias Mertmann, Linus Hötzel, Andrea Wichelhaus

**Affiliations:** 1 Department of Orthodontics and Dentofacial Orthopedics, University Hospital, LMU Munich, Goethestrasse 70, 80366 Munich, Germany; mila.janjic@med.uni-muenchen.de (M.J.R.); matthias.mertmann.extern@med.uni-muenchen.de (M.M.); linus.hoetzel@med.uni-muenchen.de (L.H.); kfo.sekretariat@med.uni-muenchen.de (A.W.); 2 Dental Clinic, Medical Care Center, Untermeitingen, Landsbergerstrasse 7, 86836 Graben, Germany; daniel1martin@bundeswehr.org

**Keywords:** orthodontics, archwire, Gummetal, nickel–titanium, NiTi, force, superelasticity, hysteresis, biomechanics

## Abstract

The aim of this study was to investigate the load deflection characteristics of Gummetal^®^ wires in comparison to nickel–titanium (NiTi) wires. Four different NiTi wires and one Gummetal^®^ archwire were analyzed in two dimensions (0.014″ (0.36 mm) and 0.016″ × 0.022″ (0.41 mm × 0.56 mm)) and in two different orientations (edgewise and ribbonwise) using three-point bending tests at T = 37 °C. Force–displacement curves were recorded and analyzed. The Gummetal^®^ 0.014″ wires exhibited higher forces compared to the NiTi wires at 2.0 mm deflection. At 1.0 mm deflection, the opposite pattern was observed. For the 0.016″ × 0.022″ Gummetal^®^ wires, the forces were within the force interval of the NiTi wires at 2.0 mm deflection. At a deflection of 1.0 mm, no residual force was measurable for the Gummetal^®^ wires. All the NiTi wires investigated showed hysteresis and a superelastic plateau. However, the Gummetal^®^ did not form a plateau, but hysteresis was present. An easier plastic deformability compared to the NiTi wires was observed for all the tested geometries.

## 1. Introduction

In orthodontics, different archwire materials, geometries, and dimensions are employed in multibracket therapy. Well-established archwire materials and alloys such as stainless steel (SS), cobalt–chromium (CoCr), beta-titanium (TMA), or superelastic nickel–titanium (NiTi) alloys exhibit specific properties, which make them suitable for different treatment tasks [1]. Besides the patient-specific factors, the criteria for selecting the most suitable archwire for the treatment task include parameters like formability, stiffness, and load deflection characteristics [1,2,3,4]. These parameters are influenced by the archwire geometry, dimensions, and mechanical properties. In the past, the use of SS wires for the leveling of malpositioned teeth often resulted in the overloading of the periodontal ligament, leading to adverse effects such as apical root resorption [5,6].

Today, superelastic NiTi wires are often used for the levelling and alignment phase [7,8,9]. NiTi alloys exhibit super elasticity [9,10,11,12] with excellent restoring forces [9], high deflectivity [9], and a low Young’s modulus [9]. The crucial clinical advantages of NiTi as compared to non-superelastic materials, are fewer wire changes, reduced “chairtime”, reduced treatment time for derotation and leveling, and decreased patient discomfort in terms of pain due to the lower force level. Although NiTi wires can be adjusted to the patients’ needs by means of cold forming or direct electric resistance heat treatment [9], bendability and formability are difficult to attain due to the high elasticity and shape memory effect [7,13]. In later treatment stages, during space closure and finishing, SS or TMA archwires are preferred due to their higher stiffness and better formability. Therefore, developing alternatives aiming to overcome the mechanical limitations of NiTi wires would provide orthodontic practitioners with more options to tailor treatments, potentially improving the efficiency and patient experience in certain aspects of orthodontic procedures, especially where bendability and formability are crucial.

Recently, wires made from Gummetal^®^ have emerged as a noteworthy development in orthodontic materials. This alloy, composed of titanium, niobium, tantalum, zirconium, and oxygen (Ti-23Nb-0.7Ta-2Zr-1.2O) [14,15,16], represents an innovative alternative, particularly for patients with nickel sensitivities [17]. Its unique composition not only ensures a nickel-free solution but also imparts good biocompatibility. Gummetal^®^ stands out for its material properties, featuring a low Young’s modulus, as well as excellent formability and low friction [15,16,17,18,19,20,21,22]. Therefore, its use has been suggested for both the initial as well as the final treatment stage [15,19,23,24]. Considering the combination of material properties, Gummetal^®^ could facilitate the application of bends at the beginning of orthodontic treatment, which is difficult to control precisely when using NiTi archwires [17]. Although the Young’s modulus of Gummetal^®^ (45 GPa) is higher compared to that of NiTi (35 GPa), it is significantly lower than that of other available alloys such as TMA (64 GPa) or SS (200 GPa) [18]. The stress–strain behavior of Gummetal^®^ has been described by the manufacturer as superelastic, although the underlying mechanisms are not fully understood and differ from the known reversible martensitic transformation found in NiTi alloys [19,25]. Some authors claim that the deformation mechanism in Gummetal^®^ occurs without phase transformation [26]. Other authors have questioned the superelasticity of the material as it shows plastic deformation, and phase transformations could be observed [17]. An available review pointed out that, based on the provided evidence, the advertised superelasticity is subject to further discussion and examination [17]. This ongoing discourse underscores the need for a better understanding of Gummetal^®^’s mechanical behavior and its implications for orthodontic applications, especially in comparison to well-established materials, such as NiTi.

Therefore, the aim of this study was to compare Gummetal^®^ wires with NiTi wires in terms of their load deflection characteristics using the standardized three-point bending test method DIN EN ISO 15841:2014 + A1:2020 [27]. This approach allowed for conclusions to be made on the clinical applicability of the different materials investigated in this study. It appears that this work represents a novel comparison between Gummetal^®^ and other NiTi-based materials.

## 2. Materials and Methods

In this study, four types of NiTi wires, Thermadent 35 °C™ (adenta^®^, Gilching, Germany), NiTi SE (dentalline^®^, Birkenfeld, Germany), BioStarter^®^/Biotorque^®^ (Forestadent^®^, Pforzheim, Germany), Titanol^®^ Superelastic (Forestadent^®^, Pforzheim, Germany), and one type of Gummetal^®^ wire (J. Morita Europe GmbH, Dietzenbach, Germany) (see Table 1) in the dimensions 0.014″ (0.36 mm) and 0.016″ × 0.022″ (0.41 mm × 0.56 mm) were evaluated by means of a 3-point bending test, according to the standard DIN EN ISO 15841:2014 + A1:2020 [27].

**Figure 1:**
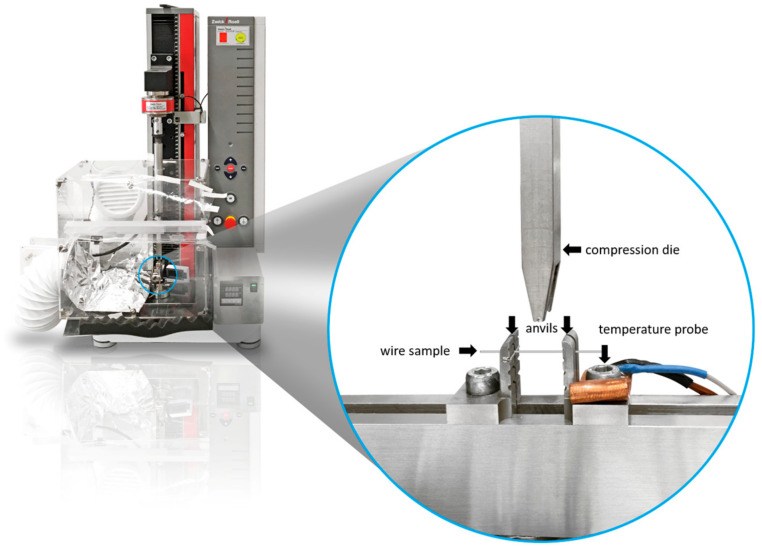
Enlarged compression dies and sample anvils of the ZwickRoell zwickiLine Z5.0 universal testing machine with temperature chamber.

For this purpose, the wires to be examined were cut to a length of 30 mm from the straight ends of conventional orthodontic wires and placed in a fixture consisting of two forks with 10 mm support spacing. A universal testing machine (zwickiLine Z5.0, ZwickRoell GmbH & Co. KG, Ulm, Germany) with a temperature chamber was used to perform measurements at T = 37 °C (Figure 1). A load cell (Xforce P, ZwickRoell GmbH & Co. KG, Ulm, Germany) with a nominal force of 20 N was attached to its crosshead. A pressure fin corresponding to DIN EN ISO 15821 [28] was mounted on a traverse, which moved at a speed of 1.25 ± 2.5 × 10^−4^ mm/min. The load was applied to a wire up to a deflection of 3.1 mm (DIN EN ISO 15821 [28]). The measured values recorded by the load cell were output as a force–displacement curve in a measuring program (testXpert II 3.41, ZwickRoell). Additionally, measured force levels during the unloading phase were extracted from the data for deflections of 2.0 mm and 1.0 mm. Each test run consisted of 3 cycles on each specimen in order to detect and visualize any possible first cycle effects or accumulation of plastic deformations. For each wire model and wire geometry, *n* = 6 samples were tested. In this series of tests, the rectangular 0.016″ × 0.022″ wires were bent “edgewise” (flat), i.e., the narrow side was loaded and “ribbonwise” (upright), i.e., the broad side was subjected to loading.

Descriptive statistics were performed and the normal distribution was tested using the Shapiro–Wilk test. The Mann–Whitney U test was used for further statistical analysis. This allowed for a pairwise comparison between the five different NiTi archwires and the Gummetal^®^ archwires. The obtained significances were then adjusted with a Bonferroni correction, which led to a significance level of αcorr. = 0.0125. SPSS 26 (IBM, Armonk, NY, USA) and Excel 2019 (Microsoft, Redmond, WA, USA) were used for the calculation [29].

## 3. Results

**Figure 2:**
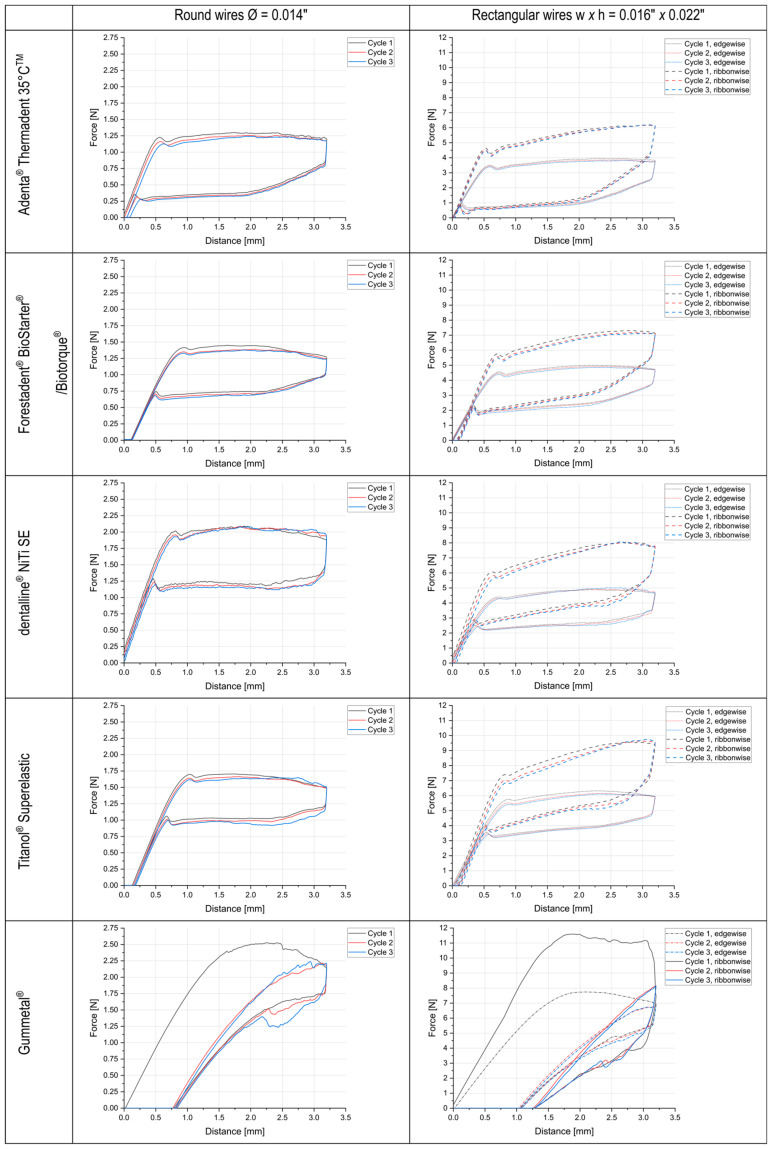
Comparison of the force–deformation diagrams from the 3-point bending test between the round and rectangular arches made of Gummetal^®^ (bottom) and NiTi round and rectangular arches. The left column displays round arches of dimension 0.014″. The right column displays rectangular arches of dimension 0.016″ × 0.022″ once edgewise and once ribbonwise. The first three force–deformation cycles are recorded in each case.

The results of the three-point bending measurements are shown in Table 2 and Table 3 and Figure 2, subdivided according to their respective cross-section or, if applicable, their directionality. Table 2 and Table 3 display the measured force levels during the unloading phase for the deflections of 2.0 mm and 1.0 mm; Figure 2 displays the corresponding force–deflection curves for all three measurement cycles.

According to DIN EN ISO 15841 [27], the unloading forces are recorded; therefore, the remaining forces for 2.0 mm deflection are shown before those for 1.0 mm deflection. For the Ø = 0.014″ round wires, Thermadent 35 °C (F_2mm_ = 0.40 N) exhibited the lowest force on recovery, with NiTi Se (F_2mm_ = 1.21 N) exhibiting the highest force level of the NiTi wires, at a remaining deflection of 2.0 mm. Compared to this, the Gummetal^®^ wires show an even higher remaining force of F_2mm_ = 1.33 N. With a deflection of 1.0 mm Ø = 0.014″, the round wires made from Gummetal^®^ (F = 0.28 N) and Thermadent 35 °C (F = 0.37 N) provide less force than the other wires (NiTi SE, Titanol Super-elastic and BioStarter). Only the Thermadent 35 °C and Gummetal^®^ wires are below the 0.5 N threshold at 1.0 mm, and Thermadent 35 °C, also at 2.0 mm of deflection.

For the rectangular 0.016″ × 0.022″ Gummetal^®^ wires, the forces for 2.0 mm deflection F_2mm_ = 3.41 N (edgewise) or F_2mm_ = 2.44 N (ribbonwise) are located within the force interval of the NiTi wires; for a deflection of 1.0 mm, no residual force was measurable. The spread of the values of the compared NiTi wires is very high and lies in the value range between F_2mm_ = 5.28 N (Titanol Super-elastic, ribbonwise) and F_2mm_ = 1.34 N (Thermadent 35 °C, ribbonwise) at 2.0 mm deflection. The edgewise directed wires exhibit lower forces at 2 mm deflection, but still, the Titanol Super Elastic wire (F_2mm_ = 3.88 N) has the highest residual forces and the Thermadent 35 °C wire the lowest (F_2mm_ = 1.02 N). Compared to this, the forces at an unloading deflection of 1.0 mm are between F_1mm_ = 0.87 N (Thermadent 35 °C, ribbonwise) and F_1mm_ = 4.43 N (Titanol Super-elastic, ribbonwise), and F_1mm_ = 0.79 N (Thermadent 35 °C, edgewise) and F_1mm_ = 3.49 N (Titanol Super-elastic, edgewise), respectively.

These force values can only be interpreted correctly if their source force–deflection curves are considered. The NiTi round wires as well as all the rectangular NiTi wires provide a curve pattern provided from the three-point bending test of the NiTi wires. The clinically relevant unloading plateau is present in all of the measured wires independent of the wire size, geometry, or directionality.

In the force–displacement diagrams of the Gummetal^®^ (see Figure 2 bottom), both the round wires (left column) and rectangular wires (right column) produce a similar curve pattern. In the first cycle, forces exhibit an initial quasi-linear rise, which can be attributed to the elastic material response. Subsequently, there is a non-linear increase in force followed by a decline, resembling the well-known curves associated with plastic deformation. The loading part of the curve is comparable to a force plateau. In contrast to this, the unloading curves do not show any plateau. It is also visible that the curves do not return to zero after unloading is completed. There seems to be a significant plastic deformation after the first cycle for all the specimens tested, ranging from about 0.5 to 1.5 mm. In the subsequent second and third deformation cycle, the curve then starts from the point of this unresolved deformation. This behavior is consistent for both the round and rectangular wires, although the rectangular wires exhibit a significantly higher maximal force, which is also the case for all the tested NiTi wires.

## 4. Discussion

The continuous advancements in orthodontic materials, such as the introduction of Gummetal^®^ wires, offer a potential alternative in addressing the challenges associated with the bendability and formability of NiTi wires. These alternatives open up new possibilities for optimizing orthodontic treatments and improving patient experiences. Therefore, this study aimed to investigate and compare Gummetal^®^ wires with NiTi wires in terms of their load deflection characteristics.

All the NiTi wires investigated in this study exhibited hysteresis in their force–displacement behavior. The degree of force hysteresis was more pronounced with an increasing cross-section, particularly noticeable in the square wires compared to the round wires, and was more apparent in the testing with the ribbonwise orientation as compared to the edgewise orientation. Although the force level at which the superelastic plateau formed differed in all the NiTi wires tested, such a plateau was observed in all of them. In contrast, the tested Gummetal^®^ wires did not form such a clear plateau. The cause of the measured force plateau in the NiTi alloys is the stress-induced martensitic phase transformation from the austenite phase to the martensite phase, the latter being thermodynamically unstable at the test temperature (T = 37 °C). The increase in the force is caused by the progression from the austenite phase to the martensite phase. In this process, an increase in the force is “consumed” by the progress of the transformation, i.e., the mechanical energy is reversibly converted into chemical energy, which is released again during the subsequent unloading. In the case of Gummetal^®^, it is also possible to assume that a phase transformation has occurred. Although the Gummetal^®^ did not exhibit a plateau, hysteresis was nevertheless present, indicating a phase transformation and the conversion of chemical energy back into mechanical energy with an energy loss upon load removal [30]. This aligns with the findings from synchrotron high-energy X-ray scattering studies, indicating that Gummetal^®^ comprises various non-uniform regions including Nb-rich B2 clusters (austenite), Nb-rich α″ nano-scale domains, and Nb-lean body centered cubic (BCC) regions. Under an applied load, these regions transform into the nanodomains of α″ and δ martensite, which contribute to the alloy’s distinct nonlinear elastic behavior [25].

**Figure 3:**
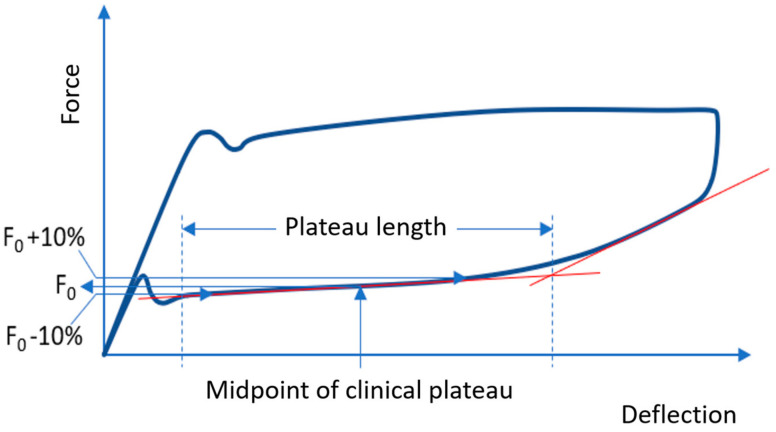
Force–deflection diagram illustrating the superelastic plateau, where F_0_ − 10% corresponds to the anterior point and F_0_ + 10% to the posterior point of the clinical plateau.

During the unloading process in the NiTi alloys, the so-called “clinical plateau” is obtained by applying the method described in Figure 3, by dividing the superelastic plateau into two sections of equal length. Afterwards, 10% is added to the corresponding force value F_0_ (for point 1: F_0_ + 10%) or subtracted (point 2: F_0_ − 10%) from this midpoint to the force value read [11]. The flatter the plateau, the greater the difference from the measured values of the deflection assigned to point 1 and point 2, respectively, and thus, the length of the clinically relevant plateau. The long and flat plateau at a low force level means a low and constant force delivery over a long distance [31,32], which is important for a smooth and efficient tooth movement. Teeth can thus be leveled and aligned, applying a constant force without having to change the wire frequently [32].

When measuring the Gummetal^®^ specimens in the three-point bending test, the force–displacement curve (Figure 2) initially showed a linear increase in the first cycle. This was followed by a section with non-linear, elastic–plastic deformation. The force–displacement curve was not proportional here, as the slope decreases. This is consistent with previous studies [33]. Subsequently, there was a region of force decrease, with an increase in deformation. Only plastic deformation took place. When the deflection was decreased, a reduction in the load occurred, accompanied by an initial decline in the force. This force drop was minimal in the case of the round wires, but quite pronounced in the case of the rectangular specimens, indicating a hysteresis process. This contradicts the investigations of Hasegawa et al., who reported hysteresis-free deformation [15,34]. With further unloading, an initial gradual decline in force was observed, indicating the beginning of a plateau. Here, a phase transformation, similar to the martensite–austenite transformation in NiTi, could take place. Ultimately, the curve exhibited a steep decline and plastic deformation remained.

This deformation mechanism has been described by several research groups, but it is supposedly not the only mechanism [25,34,35,36,37]. This observation is also not consistent with the proposed elastic deformation without stress-induced martensitic transformation [24,38,39]. To verify the deformation mechanism, further investigations, e.g., crystallographic, would be necessary.

The enhanced plastic deformability of the Gummetal wires, in comparison to the NiTi wires, was confirmed across all the tested geometries in the presented series of experiments. Based on the results, permanent deformation could be observed (see Figure 2). The elastic limit for Gummetal^®^ is known from the literature to be around ε = 2.5% [26,38], which explains the cause of the plastic strain. In the selected three-point bending test with 3.1 mm deflection at 10 mm support spacing, edge fiber strains up to 6.5% were calculated, which properly heat-treated NiTi alloys endure without permanent deformation. This was not expected for Gummetal^®^ based on the published literature’s data. To obtain a meaningful force–displacement diagram within the elastic range of Gummetal^®^, one would not be likely to test exactly according to the ISO standard. However, this series of tests was not intended to test Gummetal^®^ under the optimum conditions for this wire. Instead, the intention was to subject Gummetal^®^ to a standardized investigation, DIN EN ISO 15841:2014 + A1:2020 [27], to enable a direct comparison with other alloys, with a primary focus on the force output and the force–deformation curve.

Based on the results of this study, it can be concluded that Gummetal^®^ does not possess superelasticity or properties similar to superelastic behavior. Nevertheless, starting from the second deformation cycle, a substantial hysteresis-like deformation was observed, continuing without additional plastic strain. This behavior may well be related to the presence of an energy-consuming phase transformation in the material. However, this cannot be verified on the basis of the phenomenological investigations of the present study.

In comparison, the superelastic behavior of the NiTi wires is a clear advantage over Gummetal^®^, especially during the leveling phase. Here, the present study showed that the superelastic NiTi wires delivered significantly lower forces than Gummetal^®^ (see Table 2 and Table 3), making Gummetal less suitable for use in this initial phase. Instead, considering its application is recommended no earlier than in phases 2 and 3. The large distances of tooth movement would already have been overcome in these phases and an elongation of the wire of more than 1.0 mm is unlikely. Here, it must be critically questioned what added value Gummetal^®^ offers compared to superelastic NiTi wires. The good formability of Gummetal^®^ brings minor advantages in clinical situations where this is needed. Because of the high deformability of Gummetal^®^ [24], “stops” and “loops” can be bent in, for example, which is not necessary for NiTi wires because of the high elongation amounts.

Three-point bending tests are an accepted standard method for sampling wire parameters. They are known for their high reproducibility and decades-long utilization [11,40] in combination with a very simple test set-up, which reflects the clinical reality quite well. This test offers specimen-related characteristic values rather than material-related ones and it thus remains a valuable tool for the in vitro assessments of archwire properties. The method ensures comparability with other studies and facilitates the comprehensive evaluation of wire characteristics [11].

The presented in vitro tests serve as a valuable approximation for the clinical in vivo scenario; however, it is essential to acknowledge additional influencing factors that may not be fully captured. Factors like friction at the brackets and ligatures, which can be influenced by specific tooth malocclusions, must be taken into consideration. Additionally, variations in temperature, biochemical interactions from saliva, and varying distances between neighboring brackets or bands can alter the behavior of wires within the oral environment [41].

Furthermore, only patients with severe malocclusions require wire deflections of more than 1.0 mm and the superelastic plateau is not, to its full extent, required in many therapy cases [11].

## 5. Conclusions

Gummetal^®^ archwires show significantly higher deformability compared to NiTi archwires.

The Gummetal^®^ archwires seemed to exhibit a behavior which is similar to a force plateau, but, in contrast to the NiTi archwires, this plateau was located at very high force values and was also only present if no cold forming, such as bending, was present. It can therefore be concluded that Gummetal^®^ does not exhibit superelasticity or properties similar to superelastic behavior, as defined. However, the Gummetal^®^ archwires, while not displaying a superelastic plateau, did show hysteresis, which indicates a phase transformation in the material.

## Figures and Tables

**Table 1:** Tested wires (X) subdivided by manufacturer, trade name, and wire orientation.

Manufacturer	Trade Name	Wire Dimension		
		0.014″	0.016″ × 0.022″	
		Round	Edgewise	Ribbonwise
Adenta^®^	Thermadent 35 °C™	X	X	X
Forestadent^®^	BioStarter^®^	X		
Forestadent^®^	Biotorque^®^		X	X
dentalline^®^	NiTi SE	X	X	X
Forestadent^®^	Titanol^®^ Superelastic	X	X	X
J. Morita Europe	Gummetal^®^	X	X	X

**Table 2:** Force levels of round 0.014″ wires at 1.0 mm and 2.0 mm deflection (*n* = 6).

Model	Directionality	Force Level at 2.0 mm Deflection [N]					Force Level at 1.0 mm Deflection [N]				
		Mean (SD)	Min	Max	Md	*p*-Value	Mean (SD)	Min	Max	Md	*p*-Value
Adenta^®^ Thermadent 35 °C™	round	0.40 (0.01)	0.40	0.43	0.40	0.002	0.37 (0.02)	0.35	0.4	0.36	0.002
Forestadent^®^ BioStarter^®^	round	0.73 (0.02)	0.71	0.76	0.73	0.002	0.70 (0.02)	0.67	0.73	0.70	0.002
dentalline^®^ NiTi SE	round	1.21 (0.04)	1.15	1.26	1.21	0.009	1.19 (0.02)	1.15	1.22	1.19	0.002
Forestadent^®^ Titanol^®^ Superelastic	round	1.00 (0.04)	0.95	1.06	1.01	0.002	0.99 (0.03)	0.94	1.03	0.99	0.002
Gummetal^®^	round	1.33 (0.05)	1.24	1.37	1.34	ref.	0.28 (0.04)	0.21	0.31	0.29	ref.

(*n*) = number of samples; (SD) = standard deviation; (Min) = smallest value; (Max) = highest value; (Md) = median.

**Table 3:** Force levels of rectangular 0.016″ × 0.022″ wires in edgewise and ribbonwise directionality at 1.0 mm and 2.0 mm deflection (*n* = 6).

Model	Directionality	Force Level at 2.0 mm Deflection [N]					Force Level at 1.0 mm Deflection [N]				
		Mean (SD)	Min	Max	Md	*p*-Value	Mean (SD)	Min	Max	Md	*p*-Value
Adenta^®^ Thermadent 35 °C™	edgewise	1.02 (0.01)	1.01	1.04	1.01	0.002	0.79 (0.01)	0.77	0.81	0.79	0.002
Forestandent^®^ BioTorque^®^	edgewise	2.12 (0.4)	1.32	2.42	2.24	0.002	1.84 (0.37)	1.09	2.11	1.95	0.002
dentalline^®^ NiTi SE	edgewise	2.69 (0.1)	2.61	2.86	2.65	0.002	2.47 (0.1)	2.37	2.6	2.43	0.002
Forestadent^®^ Titanol^®^ Superelastic	edgewise	3.88 (0.08)	3.75	3.99	3.88	0.002	3.49 (0.07)	3.37	3.6	3.5	0.002
Gummetal^®^	edgewise	3.41 (0.08)	3.26	3.48	3.44	ref.	0 (0)	0	0	0	ref.
Adenta^®^ Thermadent 35 °C™	ribbonwise	1.34 (0.04)	1.28	1.38	1.34	0.002	0.87 (0.04)	0.83	0.91	0.88	0.002
Forestandent^®^ BioTorque^®^	ribbonwise	3.18 (0.1)	3.05	3.32	3.17	0.002	2.45 (0.09)	2.33	2.56	2.44	0.002
dentalline^®^ NiTi SE	ribbonwise	3.98 (0.12)	3.84	4.15	3.98	0.002	3.19 (0.1)	3.07	3.3	3.2	0.002
Forestadent^®^ Titanol^®^ Superelastic	ribbonwise	5.28 (0.1)	5.18	5.41	5.28	0.002	4.43 (0.06)	4.35	4.53	4.43	0.002
Gummetal^®^	ribbonwise	2.44 (0.33)	2.11	2.9	2.43	ref.	0 (0)	0	0.01	0	ref.

(*n*) = number of samples; (SD) = standard deviation; (Min) = smallest value; (Max) = highest value; (Md) = median.

## Data Availability

The original data set can be provided upon request.
